# Emergency left side colectomy and primary anastomosis with or without diverting stoma: a comparative study

**DOI:** 10.1007/s00384-026-05177-9

**Published:** 2026-06-25

**Authors:** Marco Ceresoli, Mauro Podda, Carola Anna Paolina Ferro, Stefano Piero Bernardo Cioffi, Alan Biloslavo, Valerio Cozza, Federico Coccolini

**Affiliations:** 1 School of Medicine and Surgery, University of Milano-Bicocca, Milan, Italy; 2 Department of General and Emergency Surgery, Fondazione IRCCS San Gerardo Dei Tintori, Via Pergolesi 33, 20900 Monza, Italy; 3 Department of Surgical Science, University of Cagliari, Cagliari, Italy; 4 Chirurgia Generale - Trauma Team ASST Grande Ospedale Metropolitano Niguarda, Milano, Italy; 5 Department of General Surgery, Cattinara University Hospital, Trieste, Italy; 6 Emergency Surgery and Trauma Unit, Fondazione IRCSS Policlinico Universitario A Gemelli Roma, Rome, Italy; 7 General, Emergency and Trauma Surgery Department, Pisa University Hospital, Pisa, Italy; 8 HPB Surgery, Hepatology and Liver Transplantation Unit, Fondazione IRCCS Istituto Nazionale Tumori, Milan, Italy; 9 Ospedale Cristo Re, Roma, Italy; 10 Department of Pathophysiology and Transplantation, University of Milan, Milan, Italy

**Keywords:** Emergency surgery, Primary anastomosis, Diverting stoma, Diverting ileostomy, Left-side perforation

## Abstract

**Background:**

Left-sided colonic perforation is a common surgical emergency. While Hartmann’s procedure has traditionally been the standard treatment, growing evidence supports primary anastomosis as a safe and effective alternative. The benefit of adding a diverting ileostomy in this scenario remains uncertain. This study aimed to evaluate the clinical impact of a diverting stoma after emergency left-sided colectomy with primary anastomosis.

**Methods:**

This was a retrospective multicentre cohort study. All consecutive adult patients undergoing emergency left-sided colectomy with primary anastomosis between 2019 and 2023 were included. Patients were divided into two groups according to the use of a diverting stoma (DS) or no stoma (NS). The primary outcome was 30-day mortality. Secondary outcomes included postoperative morbidity, anastomotic leak, reoperation, readmission, and 1-year stoma-free survival.

**Results:**

A total of 211 patients were included, of whom 75 (35.2%) received a diverting stoma. While baseline demographic characteristics and comorbidities were similar between the two cohorts, patients in the DS group presented with significantly higher rates of generalized peritonitis. Thirty-day mortality did not differ (DS 2.2% vs NS 1.3%, *p* = 0.656). Anastomotic leak occurred in 4.0% of DS and 8.8% of NS patients (*p* = 0.192). Reoperations were fewer in the DS group (4.0% vs 12.5%, *p* = 0.044). Readmission rates were higher among patients with diversion (16.2% vs 2.3%, *p* < 0.001). One-year stoma-free survival favored the NS group (96.7% vs 83.1%, *p* = 0.003).

**Conclusions:**

Diverting stoma after emergency left-sided colectomy with primary anastomosis does not influence mortality or overall morbidity but was associated with reduced reoperations related to anastomotic leaks. Diversion is associated with higher readmission rates and delayed stoma reversal, highlighting the importance of individualized, evidence-based decision-making in emergency colorectal surgery.

**Supplementary Information:**

The online version contains supplementary material available at https://doi.org/10.1007/s00384-026-05177-9.

## Introduction

Left-sided colonic perforation is one of the most frequent surgical emergencies, requiring timely intervention to control sepsis and achieve adequate source control. Historically, the standard operative approach has been the Hartmann’s procedure, consisting of sigmoid resection, rectal stump closure, and end colostomy formation, thereby avoiding primary anastomosis.

In recent years, however, several studies and randomized controlled trials, including the LADIES trial, have demonstrated that primary anastomosis is not only safe and feasible but may also offer superior outcomes compared to Hartmann’s procedure [1–7]. Despite this evidence, primary anastomosis after emergency left-sided colonic resection is still performed in fewer than one-third of patients, as reported in the Goodbye Hartmann Trial [8]. The principal concern remains the risk of anastomotic leakage, which can result in ongoing sepsis and increased morbidity and mortality.

A diverting ileostomy can be constructed to divert fecal flow and theoretically reduce the risk of anastomotic leak. However, evidence supporting this strategy in the emergency setting is limited and conflicting [9–11]. A recent meta-analysis published in 2023, including 544 patients, found no significant difference in postoperative morbidity or mortality between patients with and without a diverting ileostomy [10]. The ongoing DIVERTI2 trial aims to clarify the potential benefits of diversion following resection with primary anastomosis for perforated diverticulitis [12].

The present study aims to evaluate the clinical impact of diverting ileostomy in a large cohort of patients undergoing emergency left-sided colonic resection with primary anastomosis.

## Materials and methods

This study is reported in accordance with the STROBE (Strengthening the Reporting of Observational Studies in Epidemiology) guidelines for reporting observational studies [13]. This study is a retrospective multicentre study including six Italian high-volume emergency general surgery centers. Data about all consecutive patients diagnosed with left colonic perforation and peritonitis who underwent resective surgery between the 1 st of January 2019 and the 31 st of December 2023 were retrieved. Left colonic perforation was defined as perforation found anywhere from the distal transverse colon to the rectum, including both diffuse peritonitis or perforation with a large intra-peritoneal abscess after the failure of non-operative management. The diagnosis was based on preoperative CT scan. Patients were treated according to updated guidelines, in case of both acute diverticulitis and perforated colon cancer [14–16].

Inclusion criteria were peritonitis and/or abscess from left-sided colonic perforation; emergency sigmoid resection with primary anastomosis with or without diverting ileostomy. Patients were then subdivided into those that received a diverting stoma (DS group) and those who did not (NS group).

Exclusion criteria were patients < 18 years old, Hartmann’s procedure, diagnosis of peritonitis from right colonic perforation, patients who did not undergo resective surgery, perforations due to trauma, perforation as a complication of an elective surgery, patients that underwent damage-control surgery with open abdomen.

The primary endpoint of the study was 30-day mortality. Secondary endpoints were morbidity, reoperations and readmission, stoma-free discharge rate, and 1-year stoma-free survival.

Clinical characteristics including Clinical Frailty Scale (CFS), Charlson Comorbidity Index (CCI), and Mannheim Peritonitis Index (MPI) were collected for each patient; surgical characteristics such as timing of surgery, type, and seniority of the surgeon; surgical approach adopted. Operating surgeons were arbitrarily classified as colorectal (> 50% of working time dedicated to elective colorectal surgery), acute care (> 50% of working time dedicated to emergency general surgery and trauma), or other since in Italy there is no structured fellowships or recognized underspecialization. Senior surgeons were considered as such if > 40 years old and having performed > 50 resective colonic surgeries per year. Postoperative complications were classified using the Clavien–Dindo classification (severe complications were classified as CD > IIIa).

Given the retrospective nature of the study and the absence of a standardized protocol requiring routine radiological or endoscopic assessment of the anastomosis in all patients, asymptomatic or subclinical leaks that did not result in clinical alterations may have been missed. No standardized imaging protocol was enforced across the participating centers; diagnostic imaging (such as CT scans) was performed strictly based on clinical suspicion. Consequently, only clinically relevant anastomotic leaks—defined as a disruption of the anastomosis requiring diagnostic evaluation and/or therapeutic intervention (radiological, endoscopic, or surgical)—were considered as outcomes.

Patients were followed up by phone call with the assessment of stoma reversal rate twelve months after surgery.

Qualitative data were summarized using frequencies and percentages, quantitative data were summarized with medians and interquartile ranges. A univariate analysis was performed to determine factors associated with diverting stoma presence, Mann–Whitney *U* test was used for continuous variables while chi-squared test was used for categorical variables. Stoma-free survival was plotted using the survival curves. Multivariable models were developed with logistic regression method to assess the association between protective ileostomy and the main outcomes (mortality, severe complications, anastomotic leak, abscess, and reoperation), adjusting for patients’ clinical characteristics. Data was analyzed using SPSS Statistics (IBM Corp. Released 2023. IBM SPSS Statistics for Windows, Version 29.0.2.0 Armonk, NY: IBM Corp).

## Results

During the study period, 458 patients were retrieved: 248 underwent Hartmann’s procedure and were excluded. Characteristics and outcomes of excluded patients were summarized in supplementary Table 1. Compared with patients undergoing Hartmann’s resection, those treated with primary anastomosis were younger and had lower frailty and comorbidity scores, as well as a significantly less severe septic status; they were more often operated on during daytime hours, indicating a more favorable baseline clinical condition.

**Fig. 1:**
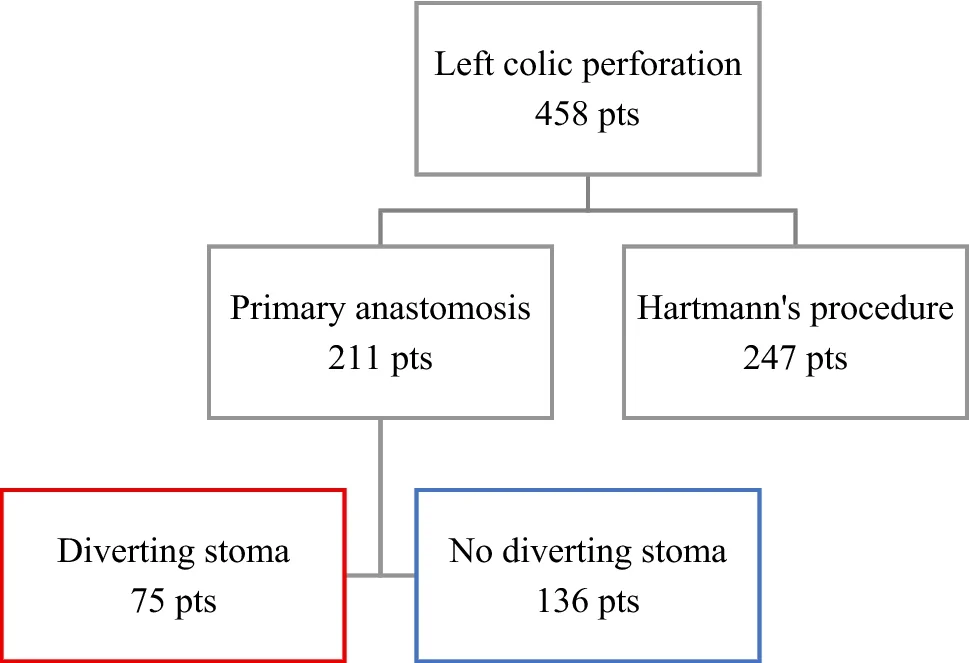
Study population

A total number of 211 patients underwent primary anastomosis and were analyzed, 75 with diverting stoma (DS, 35.2%) and 136 without diverting stoma (NS, 64.6%) (Fig. 1).

Table 1 shows the characteristics of study population. Baseline characteristics such as age, sex, BMI, and frailty scores were similar between groups. Diagnosis at presentation and time of surgery were not significantly different, though generalized peritonitis was more common in the diverting group (49.3% vs. 25.7%, *p* < 0.001).

**Table 1:** Patients characteristics

		No diverting (136)		Diverting stoma (75)		
		*N*/median	%/IQR	*N*/median	%/IQR	*p*-value
Sex	F	61	44.9%	36	48.0%	0.661
	M	75	55.1%	39	52.0%	
Age		63	(51–74)	65	(56–74)	0.100
BMI		24.45	(22.87–26.83)	25.22	(22.9–28.73)	0.220
Clinical Frailty Scale		3	(2–3)	3	(2–3)	0.319
Charlson Comorbidity index		2	(1–3)	3	(1–4)	0.083
Severe Sepsis		16	11.8%	15	20.0%	0.106
Diagnosis	Peritoneal Abscess	51	37.5%	24	32.0%	0.424
	Peritonitis	85	62.5%	51	68.0%	
Time of surgery	Morning	66	48.5%	27	36.0%	0.116
	Afternoon	49	36.0%	29	38.7%	
	Night	21	15.4%	19	25.3%	
Weekend or holiday		30	22.1%	20	26.7%	0.451
Surgeon speciality	Acute care surgeon	83	71.6%	33	28.4%	0.020
	Colorectal surgeon	41	60.3%	27	39.7%	
	Other	12	44.4%	15	55.6%	
Operating surgeon age > 40		87	64.0%	54	72.0%	0.236
Operating time (minutes)		225	(180–280)	265	(203–325)	0.029
MIS approach		71	52.2%	27	36.0%	0.024
Succesful laparoscopy		40	29.4%	12	16.0%	0.030
Drain		98	72.1%	58	77.3%	0.404
Peritonitis extension	Localized	101	74.3%	38	50.7%	< 0.001
	Diffused/generalized	35	25.7%	37	49.3%	
Kind of exudate	Clear	45	33.1%	18	24.0%	0.385
	Purulent	80	58.8%	50	66.7%	
	Fecal	11	8.1%	7	9.3%	
Mannheim peritonitis Index		15	(10–20)	16	(12–21)	0.004

Acute care surgeons performed diverting stoma less frequently than colorectal and other surgeons (28.4% vs. 39.7% vs 55.6%, *p* = 0.02). Patients treated without diverting stoma were more commonly treated with laparoscopy (29% vs 16%, *p* = 0.03).

Table 2 depicts study outcomes. Mortality did not differ significantly between the two study groups (2.2% vs. 1.3%, *p* = 0.656). The overall complication rate was similar between the two groups (58.7% vs. 47.1%, *p* = 0.106) with severe complications more common in the non-diverting stoma group (16.9% vs. 8%, *p* = 0.072) even if not significantly.

**Table 2:** Study outcomes

		No diverting (136)		Diverting stoma (75)		*p*-value
		N/median	%/IQR	N/median	%/IQR	
30-day mortality		3	2.2%	1	1.3%	0.656
Any complication		64	47.1%	44	58.7%	0.106
Clavien-Dindo Grade	0	72	52.9%	31	41.3%	0.008
	I	24	17.6%	14	18.7%	
	II	14	10.3%	23	30.7%	
	IIIa	3	2.2%	3	4.0%	
	IIIb	12	8.8%	1	1.3%	
	IVa	7	5.1%	2	2.7%	
	IVb	1	0.7%	0	0.0%	
	V	3	2.2%	1	1.3%	
Major complications		23	16.9%	6	8.0%	0.072
Reintervention		17	12.5%	3	4.0%	0.044
	Anastomotic leak	12	8.82%	3	4.0%	
	Bleeding	3	2.20%	0	0%	
	Other	2	1.47%	0	0%	
SSI within 30 gg		11	8.1%	8	10.7%	0.531
Abdominal Abscess		12	8.8%	6	8.0%	0.838
anastomotic leak		12	8.8%	3	4.0%	0.192
Respiratory infections		11	8.1%	7	9.3%	0.757
Renal faliure		1	0.7%	3	4.0%	0.096
Cardiovascular complications		7	5.1%	2	2.7%	0.393
Urinary infections		1	0.7%	4	5.3%	0.036
stoma related complications		1	0.7%	1	1.3%	0.668
ICU admission		15	11.0%	12	16.0%	0.301
ICU days		2	(1–7)	2	(1–3)	0.817
N. of ventilation days		1	(0–1)	1	(0–2)	0.382
LOS (days)		12	(8–17)	13	(10–18)	0.106
Stoma at discharge	None	125	91.9%	0	0.0%	0.001
	End stoma	10	7.4%	0	0.0%	
	Diverting stoma	1	0.7%	75	100.0%	
30-day Readmission		3	2.3%	12	16.2%	< 0.001
Readmission due to stoma complications		0	0.0%	2	16.6%	0.544
Lost at follow-up		9	6.6%	4	5.3%	0.689
1-year recanalization		4	50.0%	59	83.1%	0.02
Time to recanalization (months)		7.93	(7.67–8.50)	4.57	(3.43–6.50)	0.016
1-year stoma free survival		120	96.7%	59	83.1%	0.003

Clinically relevant anastomotic leak was detected in 8.8% of patients without diverting stoma compared to 4.0% in patients with diverting stoma (*p* = 0.192). Reintervention occurred more often in the no-diverting group (12.5% vs. 4.0%, *p* = 0.044). Among postoperative morbidity patients with diverting stoma have a higher rate of urinary infections (0.7% vs 5.3% *p* = 0.036). No differences in length of stay were noticed (13 vs. 12 days, *p* = 0.106).

At multivariable analysis, the presence of a diverting ileostomy was not significantly associated with mortality, severe complications, abscess formation, or anastomotic leak. Conversely, diverting ileostomy was associated after adjustment with a significantly reduced risk of reintervention (aOR 0.07, 95% CI 0.005–0.883; *p* = 0.039) (Table 3).

**Table 3:** Multiple regression analysis

	Mortality		Severe complications		Abscess		Anastomotic leak		Reintervention	
	aOR (95%CI)	*p* value	aOR (95%CI)	*p* value	aOR (95%CI)	*p* value	aOR (95%CI)	*p* value	aOR (95%CI)	*p* value
Diverting stoma	0.314 (0.031–3.097)	0.321	0.284 (0.052–1.542)	0.144	2.08 (0.574–7.543)	0.264	0.179 (0.018–1.709)	0.135	0.07 (0.005–0.883)	0.039
Sex (M)	0.908 (0.321–2.566)	0.855	2.094 (0.446–9.826)	0.348	1.404 (0.382–5.157)	0.608	1.856 (0.306–11.224)	0.500	2.164 (0.392–11.945)	0.375
Age	1.055 (1.003–1.11)	0.035	0.935 (0.88–0.993)	0.031	0.972 (0.914–1.034)	0.375	0.95 (0.884–1.022)	0.173	0.954 (0.896–1.015)	0.142
Clinical Frailty Scale	1.507 (1.131–2.008)	0.004	1.269 (0.79–2.039)	0.323	1.269 (0.738–2.182)	0.387	0.958 (0.555–1.652)	0.878	1.184 (0.745–1.881)	0.473
Charlson comorbidity index	1.227 (0.975–1.543)	0.080	2.102 (1.251–3.531)	0.005	1.017 (0.615–1.682)	0.945	1.387 (0.821–2.345)	0.220	1.815 (1.099–2.995)	0.019
Severe sepsis	1.478 (0.956–2.684	0.059	3.002 (0.584–15.425)	0.187	0.788 (0.143–4.325)	0.784	2.032 (0.292–14.118)	0.473	0.971 (0.134–7.027)	0.977
Abscess vs. diffuse peritonitis	0.443 (0.113–1.732)	0.242	0.685 (0.18–2.608)	0.579	0.564 (0.158–2.015)	0.378	0.52 (0.104–2.587)	0.425	0.749 (0.181–3.099)	0.691
Colorectal surgeon (vs acute care)	0.896 (0.457–1.758)	0.852	11.464 (1.729–75.972)	0.011	0.584 (0.061–5.535)	0.639	24.03 (2.833–203.817)	0.003	9.594 (1.108–83.045)	0.040
Mannheim peritonitis index	1.11 (1.039–1.185)	0.001	0.938 (0.838–1.05)	0.270	0.983 (0.879–1.099)	0.770	1.004 (0.87–1.158)	0.952	0.96 (0.844–1.092)	0.535

At discharge, all patients in the diverting group had a stoma, while only 8.1% of the no-diverting group were discharged with a stoma (*p* = 0.001).

Thirty-day readmission was significantly higher in the diverting group (16.2% vs. 2.3%, *p* < 0.001), though readmission due to stoma-related complications was infrequent (16.6% of the readmissions; other reasons were non-abdominal infections (41.6%), bleeding (16.6%), pulmonary embolism (16.6%), and evisceration (8.33%)).

Nine patients (6.6%) in the NS group and 4 (5.3%) in the DS group were lost at follow-up.

Fifty-nine patients (83.1%) in the DS group had stoma closure within 1 year after a median time of 4.57 months. Among the NS group patients discharged with a stoma, 4 (50%) were recanalized after a median of 7.93 months.

**Fig. 2:**
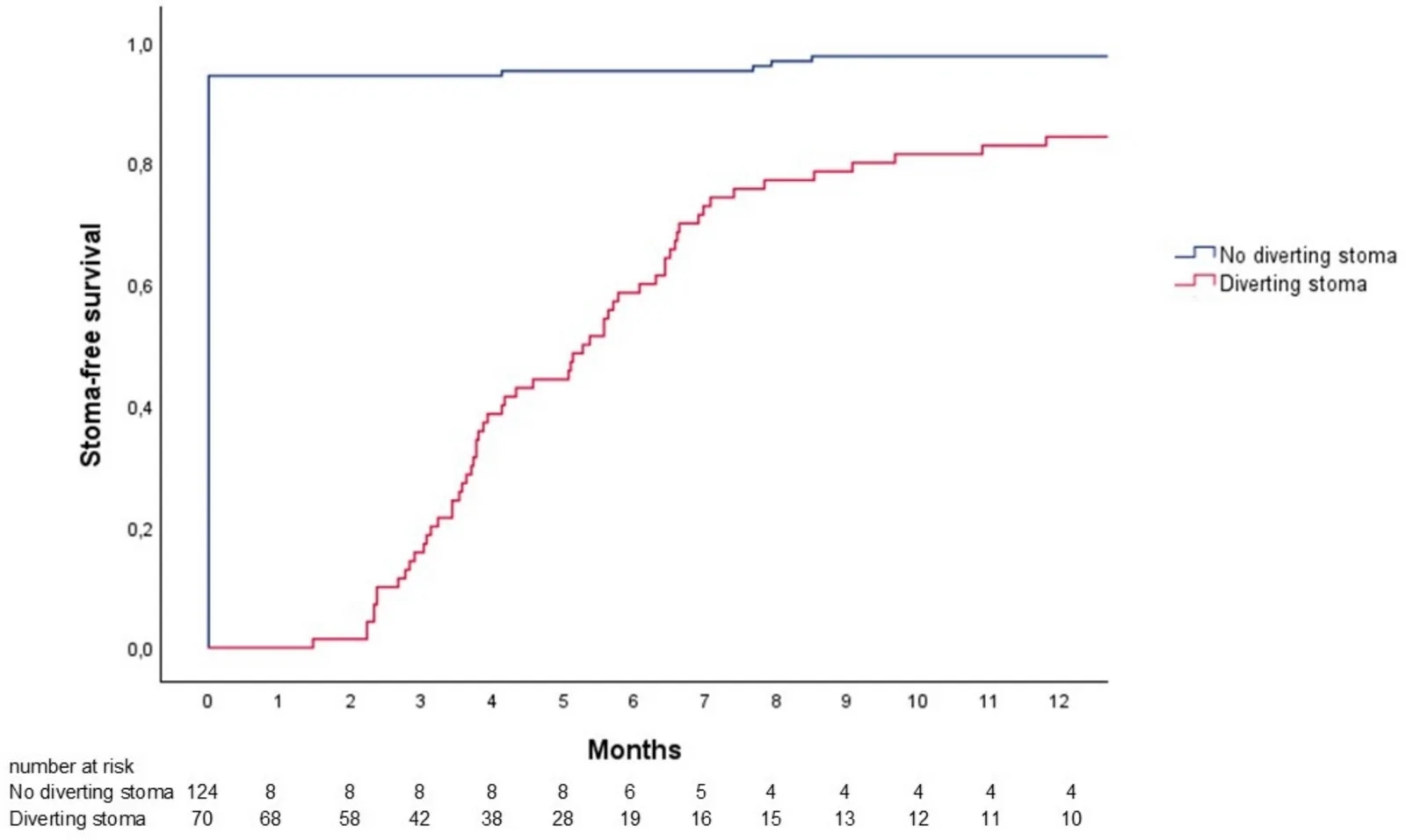
12-months stoma-free survival

One-year stoma-free survival was higher in the no-diverting group (96.7% vs. 83.1%, *p* = 0.003) (Fig. 2).

## Discussion

This study compared outcomes of patients undergoing emergency left-sided colectomy with primary anastomosis, with or without a diverting stoma. No significant differences in morbidity or mortality were observed between the two groups. The incidence of clinically relevant anastomotic leak was lower—though not completely eliminated—among patients with a diverting stoma, who also required fewer reoperations compared to those without diversion. However, patients in the diverting stoma group had higher readmission rates and a longer persistence of stoma at 12-month follow-up.

Baseline characteristics were comparable between the two groups, with no major clinical or demographic differences observed in terms of age, comorbidities, or frailty scores. This suggests that both cohorts were broadly similar at presentation, supporting the internal validity of the comparison. Nevertheless, as the decision to perform primary anastomosis and moreover a diverting stoma was made intraoperatively at the surgeon’s discretion, selection bias cannot be entirely excluded.

Anastomotic leakage remains one of the most feared complications after emergency colorectal surgery. In this cohort, the use of a diverting stoma was associated with a reduction in clinically relevant anastomotic leak (4.0% vs. 8.8%), although this difference did not reach statistical significance. This trend is consistent with previous reports suggesting that diversion may mitigate the clinical consequences of a leak rather than completely prevent it [10]. Indeed, the presence of a diverting stoma does not eliminate the risk of subclinical or contained leaks, as diversion only reduces mechanical and bacterial load at the anastomotic site, as demonstrated by the similar abdominal abscess formation rate.

Within the limitations of this retrospective analysis, the presence of a diverting stoma was associated with fewer reinterventions, a finding that supports the hypothesis that diversion may facilitate more conservative, nonoperative management of anastomotic leaks when they occur. In the presence of a stoma, minor leaks or localized abscesses may often be treated nonoperatively with antibiotics, percutaneous drainage, or endoscopic interventions. This observation was also confirmed by the multivariable regression analysis, in which, after adjustment for clinical characteristics and potential confounders, diverting stoma was significantly associated with fewer reoperations. Despite this advantage, mortality did not differ significantly between groups. This finding indicates that while diversion reduces the need for reoperation, it does not necessarily translate into improved survival. Moreover, postoperative length of stay was similar between the two groups.

The overall complication rate was comparable between groups, though severe complications (Clavien–Dindo > IIIa) were less frequent in the DS group, due to the less reintervention rate, even if statistically not significant. However, specific stoma-related complications must not be overlooked. In our study, urinary infections were more frequent among DS patients, likely reflecting dehydration and electrolyte imbalances associated with high-output stomas. These issues can also precipitate acute kidney injury and may facilitate readmission, that was higher in the DS group. It should be noted that the higher readmission rate could be interpreted also as a reflection of baseline disease severity and the profound physiological toll of sepsis rather than the diversion itself. Therefore, while a diverting stoma may offer protection against anastomotic-related reintervention, it introduces its own morbidity spectrum that must be considered in clinical decision-making, above all in frail patients, as reported by literature [17–19].

Follow-up data revealed important differences between the two groups. All patients in the DS group were discharged with a stoma, while the vast majority of those in the NS group left the hospital stoma-free. Although most DS patients underwent reversal within one year, a considerable proportion (16.9%) still had a stoma at one year, compared to only 3.9% in the NS group.

The lower 12-month stoma-free survival observed in patients with diverting ileostomy, despite comparable baseline comorbidities, may be partially explained by factors unrelated to surgical complexity. In real-world practice, logistical barriers and limited institutional resources for elective surgery may significantly delay or preclude stoma reversal, even when technically straightforward. Additionally, a proportion of patients may be lost to follow-up or decline further surgery once the acute phase has resolved. The need for stoma closure represents an additional operation with inherent morbidity, hospital stay, and economic burden for national health systems [20, 21]. Furthermore, living with a stoma has significant quality-of-life implications, affecting body image, social functioning, and psychological well-being [22, 23]. These long-term considerations must be carefully weighed when deciding for diversion in the emergency setting.

Surgeon background could have played a role in the decision to perform diversion. Acute care surgeons were less likely to construct a diverting stoma compared to colorectal or general surgeons. This difference likely reflects both technical expertise and philosophical attitudes toward risk management. Acute care surgeons, often operating in time-critical, resource-limited settings, may prioritize source control and rapid recovery, avoiding additional procedures that could complicate postoperative management. Conversely, colorectal surgeons, more accustomed to elective anastomotic surgery, may favor diversion in high-risk cases to mitigate postoperative leak consequences., as suggested also by previous research [24].

The main limitations of this study are its retrospective design and the inherent selection bias related to the non-randomized allocation of a diverting stoma, which was left entirely to the operating surgeon’s discretion. Crucially, our cohort is subject to confounding by indication; patients in the diverting stoma group presented with higher prevalence of generalized peritonitis and elevated Mannheim Peritonitis Index (MPI) scores compared to the no-diverting group. Consequently, these high-risk clinical profiles and unmeasured intraoperative factors may have independently influenced postoperative outcomes—such as complications and readmission rates—irrespective of the independent effect of surgical diversion itself. Although our multicenter design and multivariable regression analysis attempt to control for these confounding clinical characteristics, selection bias cannot be completely eliminated, and the results should be interpreted within this context.

Moreover, despite this being one of the largest studies on this topic to date, the sample size may have been insufficient to detect small but clinically relevant differences in leak and mortality rates, with a consequent risk of an underpowered study.

## Conclusions

Emergency left-sided colectomy with primary anastomosis and a diverting stoma did not result in better overall perioperative outcomes compared with primary anastomosis without a diverting stoma, with similar morbidity and mortality rates. Within the strict boundaries of this retrospective design, the presence of a diverting stoma was associated with a lower rate of reinterventions related to anastomotic leaks and a higher readmission rate and with a substantial proportion of patients still having a stoma at 12 months of follow-up. However, due to confounding by indication and the inherent selection bias of non-randomized treatment allocation, these findings must be interpreted with caution and viewed as associative rather than causal. Prospective studies and ongoing randomized trials are expected to provide further evidence to better define the indications for diverting stoma after primary anastomosis in emergency colorectal surgery.

## Supplementary Information

Below is the link to the electronic supplementary material.

ESM 1 Supplementary Material (DOCX 24.3 KB)

## Data Availability

Data are available under request to the corresponding author.
