# Supplementary material for: Emergency left side colectomy and primary anastomosis with or without diverting stoma: a comparative study
Source: Int J Colorectal Dis. 2026 Jun 25;41(1):145. doi: 10.1007/s00384-026-05177-9 (PMC13553671; doi:10.1007/s00384-026-05177-9)
Supplement: Supplementary file 1 [file 384_2026_5177_MOESM1_ESM.docx]

|  |  | Hartmann | |  | Resection + primary anastomosis | |  | p-value |
| --- | --- | --- | --- | --- | --- | --- | --- | --- |
|  |  | N | % |  | N | % |  |  |
| Sex | F | 151 | 60,9% |  | 97 | 46,0% |  | 0,001 |
|  | M | 97 | 39,1% |  | 114 | 54,0% |  |  |
| Age | | 75 | (64-84) |  | 63 | (52-74) |  | <0,001 |
| BMI | | 25 | (22,27-27,7) |  | 25 | (22,88-27,77) |  | 0,572 |
| Clinical Frailty Scale | | 4 | (3-6) |  | 3 | (2-3) |  | <0,001 |
| Charlson Comorbidity index | | 4 | (2-6) |  | 2 | (1-3) |  | <0,001 |
| Septic status | None | 133 | 53,6% |  | 179 | 84,8% |  |  |
|  | Severe Sepsis | 70 | 28,2% |  | 30 | 14,2% |  |  |
|  | Septic Shock | 45 | 18,1% |  | 2 | 0,9% |  |  |
| Diagnosis | Peritoneal Abscess | 29 | 11,7% |  | 75 | 35,5% |  | <0,001 |
|  | Peritonitis | 219 | 88,3% |  | 136 | 64,5% |  |  |
| Time of surgery | Morning | 39 | 15,7% |  | 93 | 44,1% |  | <0,001 |
|  | Afternoon | 102 | 41,1% |  | 78 | 37,0% |  |  |
|  | Night | 107 | 43,1% |  | 40 | 19,0% |  |  |
| Weekend or Holiday |  | 78 | 31,5% |  | 50 | 23,7% |  | 0,065 |
|  |  |  |  |  |  |  |  |  |
| operatorspeciality | Acute care surgeon | 138 | 55,6% |  | 116 | 55,0% |  | 0,272 |
|  | Colorectal surgeon | 67 | 27,0% |  | 68 | 32,2% |  |  |
|  | Other | 43 | 17,3% |  | 27 | 12,8% |  |  |
| Operating surgeon age>40 |  | 156 | 62,9% |  | 141 | 66,8% |  | 0,381 |
| Operating Time (minutes) | | 165 | (127-210) |  | 240 | (187-300) |  | <0,001 |
| Succesful laparoscopy |  | 8 | 3,2% |  | 52 | 24,6% |  | <0,001 |
| Drain |  | 193 | 77,8% |  | 156 | 73,9% |  | 0,331 |
| Peritonitis extension | localized | 76 | 30,6% |  | 139 | 65,9% |  | <0,001 |
|  | Diffused/generalized | 172 | 69,4% |  | 72 | 34,1% |  |  |
| Kind of Exudate | clear | 32 | 12,9% |  | 63 | 29,9% |  | <0,001 |
|  | Purulent | 153 | 61,7% |  | 130 | 61,6% |  |  |
|  | Fecal | 63 | 25,4% |  | 18 | 8,5% |  |  |
| Mannheim peritonitis Index | | 22 | (17-28) |  |  |  |  | <0,001 |
| 30-day mortality |  | 49 | 19,8% |  | 4 | 1,9% |  | <0,001 |
| Any complication |  | 179 | 72,2% |  | 108 | 51,2% |  | <0,001 |
| Clavien-Dindo Grade | 0 | 69 | 27,8% |  | 103 | 48,8% |  | <0,001 |
|  | I | 24 | 9,7% |  | 38 | 18,0% |  |  |
|  | II | 71 | 28,6% |  | 37 | 17,5% |  |  |
|  | IIIa | 7 | 2,8% |  | 6 | 2,8% |  |  |
|  | IIIb | 12 | 4,8% |  | 13 | 6,2% |  |  |
|  | IVa | 11 | 4,4% |  | 9 | 4,3% |  |  |
|  | IVb | 5 | 2,0% |  | 1 | 0,5% |  |  |
|  | V | 49 | 19,8% |  | 4 | 1,9% |  |  |
| Major complications |  | 77 | 31,0% |  | 29 | 13,7% |  | <0,001 |
| Reintervention |  | 30 | 12,1% |  | 20 | 9,5% |  | 0,662 |
| SSI within 30 gg |  | 34 | 13,7% |  | 19 | 9,0% |  | 0,116 |
| Abdominal Abscess |  | 21 | 8,5% |  | 18 | 8,5% |  | 0,981 |
| anastomotic leak |  | 6 | 2,4% |  | 15 | 7,1% |  | 0,017 |
| Respiratory infections |  | 37 | 14,9% |  | 18 | 8,5% |  | 0,036 |
| Renal faliure |  | 25 | 10,1% |  | 4 | 1,9% |  | <0,001 |
| Cardiovascular complications |  | 41 | 16,5% |  | 9 | 4,3% |  | <0,001 |
| Urinary infections |  | 14 | 5,6% |  | 5 | 2,4% |  | 0,079 |
| stoma related complications |  | 5 | 2,0% |  | 2 | 0,9% |  | 0,352 |
| ICU admission |  | 114 | 46,0% |  | 27 | 12,8% |  | <0,001 |
| ICU days | | 3 | (2-8) |  | 2 | (1-4) |  | 0,009 |
| N. of ventilation days | | 2 | (1-5) |  | 1 | (0-1) |  | 0,007 |
| LOS (days) | | 14 | (8-25) |  | 12 | (9-18) |  | 0,134 |
| Stoma at discharge | None | 0 | 0,0% |  | 126 | 59,7% |  | <0,001 |
|  | End stoma | 248 | 100,0% |  | 10 | 4,7% |  |  |
|  | Diverting stoma | 0 | 0,0% |  | 75 | 35,5% |  |  |
| 30-day Readmission |  | 15 | 7,6% |  | 15 | 7,2% |  | 0,354 |
| Readmission due to stoma complications | | 0 | 0,0% |  | 2 | 12,5% |  | 0,219 |

Supplementary table: Hartmann resection vs primary anastomosis
